# Insights from tuberose farmers: A survey study in Jashore District, Bangladesh

**DOI:** 10.1371/journal.pone.0302841

**Published:** 2024-05-03

**Authors:** Rabita Zaman, Abu Noman Faruq Ahmmed

**Affiliations:** Department of Plant Pathology, Sher-e-Bangla Agricultural University, Dhaka, Bangladesh; Sheikh Hasina National Institute of Burn & Plastic Surgery, BANGLADESH

## Abstract

To obtain information on the cultivation of tuberoses in Bangladesh’s Jashore district, a study has been carried out to evaluate the economic and social standing of cultivators and examine the methods employed in production and farmers’ attitudes towards field disease. The majority of surveyed farmers were men, aged between 30 and 40 years. Only 7.41% had a high level of education, and most had less than five years of experience in tuberose cultivation. On an annual basis, the total production cost amounted to Tk. 27,200 (bigha/year), yielding a net profit of Tk. 1,20,000 (bigha/year). 31.48% of farmers expressed the belief that diseases affecting tuberose originated from contaminated planting materials. A significant proportion (64.81%) of farmers used PGR to combat diseases affecting tuberose production. Government Agriculture Officers played a crucial role in assisting farmers with knowledge and guidance. Yet, most participants highlighted that the key to preventing diseases lies in the effective application of pesticides. The findings of this study can guide policymakers in implementing measures to enhance tuberose production and fortify the floriculture industry in Bangladesh.

## Introduction

Flowers represent beauty and purity, form a noteworthy sector in various developing and least developed countries, acknowledged for their potential for considerable expansion. Allegedly, the cultivation of flowers generates returns that are 3 to 5 times higher than rice cultivation and 1.5 to 2 times higher than the cultivation of vegetables [1]. Tuberose is well-known for its calming fragrance, possessing the ability to rejuvenate the mind and alleviate stress. Presently, it is widely employed in the production of perfumes and essential oils [2]. Cultivating flowers, especially in the Jashore district of Bangladesh, generates considerably greater profits compared to rice and vegetables, spanning approximately 9,000 hectares of land and engaging over 4,000 farmers, this sector supports the livelihoods of around 200,000 individuals, highlighting its noteworthy profitability. The flower production in Jashore district alone fulfills 70% of the nation’s demand for flowers. The domestic market is estimated at Tk. 300 crore (approximately 273 crore USD), whereas international transactions contribute significantly more, amounting to Tk. 16,000 crore (approximately 145.6 crore USD). In the fiscal year 2009–2010, the country gained Tk. 60 crore (approximately 0.546 crore USD) from the export of flowers, underscoring the profitability of commercial flower production and its substantial role in creating employment opportunities [3]. The global flower market generates approximately $36 billion in sales annually, presenting Bangladesh with the opportunity to seize a share of $500 million in sales potential [4].

Floriculture in Bangladesh took root in the late seventies when forward-thinking farmers began to concentrate on the cultivation of tuberose. The cost of tuberose production was 26% more than that of bananas and 12% higher than papaya. The gross margin from tuberose cultivation in Jashore was higher than that in Chuadanga. They were anticipated at Tk. 6,81,637, the gross return per hectare reveals a significant gross margin of Tk. 5,52,354. Chuadanga witnessed heightened returns in tuberose cultivation, recording a net profit of Tk. 4,80,876 per hectare, mainly attributed to its comparatively lower production costs. The cultivation of tuberose resulted in net returns that exceeded the banana by 65% and surpassed the papaya by 71%. Evident in the benefit-cost ratios of 5.27 against total costs and 3.39 against variable costs, it showcases robust profitability [5]. The total cost of tuberose production per hectare was Tk. 369,350, comprising variable costs of Tk. 220,720 (59.76%) and fixed costs of Tk. 148,630 (40.24%), with labor, seed, and fertilizer significantly influencing production expenses, exacerbated by the substantial application of fertilizer and insecticide, and pesticide sprays by farmers [6]. Producers navigated a marketing channel characterized by a variety of intermediaries and direct engagement with customers. Outperforming petty traders (Tk. 8.61), traders (Tk. 17.35), and wholesalers (Tk. 22.02), retailers attained the highest net marketing margin at Tk. 146.39. In harvesting season, producers sold at Tk. 0.52 per unit, whereas consumers acquired the product at Tk. 0.74. After harvesting, the purchase cost experienced an increase to Tk. 2.49 as well as consumers acquired it at Tk. 2.63 [7]. Flower farmers encounter significant economic and technical hurdles, such as the lack of parent plants and their increased costs, the expenses associated with fertilizers and pesticides, and inadequate technical expertise and training to address pest and disease attacks. Furthermore, problems related to the market include an unstructured market, insufficient transportation and communication systems, diminished market prices, and an absence of market data. The cultivation of tuberose encounters challenges from a variety of insect pests and fungal diseases. Diseases play a significant role in agricultural crop losses, contributing to approximately 13% of losses worldwide [8].

The objective of this study is to gather insights from tuberose farmers in the Jashore district of Bangladesh through a comprehensive survey, aiming to understand their cultivation practices, challenges, and perspectives on tuberose farming.

## Materials and methods

### Study site

The study covered 18 villages located in the Jhikargacha upazila of Jashore district. The site, situated at a longitude of 89°08′E and latitude of 23°06′N, was positioned at an elevation of 9 meters above sea level. It is part of the agro-ecological region identified as the "High Ganges River Floodplain" (Agro-Ecological Zone 11).

### Sample size

The objective of this research was to assess the status of tuberose cultivation in the Jashore district of Bangladesh. To achieve this, 54 tuberose farmers were surveyed to gather insights into the current practices and conditions of tuberose farming in the region. In particular, the survey targeted 18 villages spanning 4 Unions in the Jhikargacha upazila of Jashore district. Within each village, 3 farmers were selected to participate as respondents in the study (Table 1).

**Table 1:** Experimental sites under survey at Jhikargacha Upazila in Jashore District of Bangladesh.

Unions	Villages	Number of field/farmer
Godkhali	Godkhali	3
	Patuapara	3
	Sadirali	3
	Mothbari	3
	Dhalipara	3
Panisara	Panisara	3
	Syedpara	3
	Krishnachandrapur	3
	Kuliya	3
	Gaburapur	3
Navaron	Hariya	3
	Baisa	3
	Sharifpur	3
	Chandpur	3
	Mathuapara	3
Nirbashkhola	Nirbashkhola	3
	Shiorda	3
	Kanarali	3
**Total: 4**	**Total: 18**	**Total: 54**

### Data collection

The information for this study was gathered via a survey questionnaire administered through in-person interviews. It is important to note that this study received ethical approval from the Plant Pathology Department of Sher-e-Bangla Agricultural University on the 10^th^ of January, 2019. Data collection took place from February 2nd to March 5th, 2019. During this period, researchers approached eligible participants and requested their voluntary participation in the study. Participation in the study was entirely voluntary, and participants had the right to decline or withdraw from participation at any time without consequence. All data collected was anonymized and handled confidentially following the ethical guidelines outlined in the approved research protocol. Farmers participated in interviews, responding to questionnaires to share information. The questionnaire, our primary data collection tool, underwent meticulous crafting and pre-testing in Dhaka and Manikganj districts prior to the survey’s official launch. The data pertaining to tuberose cultivation were then collected using the rigorously pretested and well-structured questionnaire. Information and data regarding tuberose cultivation were acquired through a questionnaire focusing on the subsequent subjects:

1. Details about the Land
2. Time and Area of Cultivation
3. Ratio of Benefits to Costs
4. Expenses for Pest Management
5. Application of Fertilizers
6. Presence of Insects
7. Incidence of Diseases
8. Infestation of Weeds
9. Interrelation of Insects, Diseases, and Weeds
10. Measures Taken to Address Pest Infestation
11. Primary Challenges in tuberose cultivation
12. Recommendations for Disease Management

### Data analysis

The survey data underwent analysis using statistics. The information gathered from the fields underwent dedicated processing using the computer-based software STATISTIX 10.

## Results

### Socio-economic status of the survey

Conducted in the Jhikargacha upazila of Jashore district, the on-site survey involved 54 tuberose farmers, with 90.74% identified as male and the remaining 9.26% as female (Table 2). This comprehensive study encompassed the demographic landscape of 54 farmers in the Jashore district of Bangladesh. A substantial portion of farmers (50.00%) fell within the 30 to 40 years’ age bracket, with 22.22% aged between 40 and 50, and 14.82% below 30 years. Only 12.96% were above 50 years old. Examining educational backgrounds, the majority (37.04%) of surveyed tuberose farmers in Jashore completed classes I-V, securing the top position in the educational distribution. A smaller percentage (5.55%) attained Honors level, 7.41% were postgraduates, and a notable portion (18.52%) were found to be illiterate.

**Table 2:** The socio-demographic profile of the participants in the present survey.

Gender			Age of tuberose growers			Education of tuberose farmers			Land utilization under tuberose			Duration of tuberose cultivation		
Gender	No. respondents	Response(%)	Age range	No. respondents	Response (%)	Education range	No. respondents	Response (%)	Land Size	No. respondents	Response (%)	Year	No. respondents	Response (%)
Male	49	90.74	<30	8	14.82	Illiterate	10	18.52	<2	15	27.78	<5	47	87.04
Female	5	9.26	30–40	27	50.00	Class 1–5	20	37.04	2–5	28	51.85	5–10	5	9.26
**Total**	**54**	**100**	40–50	12	22.22	Class 6-SSC	12	22.22	5–8	8	14.81	10–15	2	3.70
			50<	7	12.96	HSC	5	9.26	8<	3	5.56	15<	0	0
			**Total**	**54**	**100**	Honors	3	5.55	**Total**	**54**	**100**	**Total**	**54**	**100**
						Masters	4	7.41						
						**Total**	**54**	**100**						

In terms of land utilization, a majority of Jashore farmers (51.85%) utilized land ranging from 2 to 5 bigha, ranking first in land utilization, while the lowest number (5.56%) utilized land below 8 bigha. Regarding tuberose cultivation experience, farmers in Jashore exhibited varying durations of engagement. A small percentage (3.70%) possessed over 10 years of experience, ranking last, whereas the majority (87.04%) cultivated tuberose for 5 to 10 years, securing the top position. Approximately 9.26% of farmers were relatively new to tuberose cultivation, with less than 5 years of experience.

The survey indicates that the majority of tuberose growers in the region are male, aligning with the broader scenario in Bangladesh, although there is an emerging presence of females in the floriculture industry. Notably, half of the respondents fall within the 30–40 years’ age group, indicating a mature and decisive demographic. Unfortunately, the survey highlights a concerning low literacy rate among tuberose growers

### Insights into tuberose farming practices

Tuberose cultivation revolves around the utilization of bulbs as the primary planting material. In a survey encompassing 54 farmers, the acquisition of bulbs from diverse sources was noted. Significantly, approximately 70.37% of tuberose farmers sourced bulbs from their own preservation. Furthermore, bulbs were obtained from importers (18.52%), neighbors (5.56%), local markets (3.70%), and seed dealers (1.85%) (Table 3). The survey underscores the critical role of organic manure in flowering and bulb development, cautioning against excessive use, which may lead to excessively tall and slender flower spikes.

**Table 3:** Overview of tuberose cultivation and cost-profit analysis in Jashore.

Farmers’ opinion on the source of planting materials used for tuberose cultivation			Cost involved in pest management of tuberose cultivation				Cost involvement in tuberose cultivation (Bigha/year) at Jashore		Benefit-cost analysis of tuberose (Bigha/year)			
Source of tuberose planting materials	No. of respondents	Response%	Cost/Bigha/ Year (TK)			Other pest control cost Tk./Bigha/Year	Cost involvement	Taka/Bigha	Production (sticks/year)	Price (Tk/stick)	Total cost Tk (Bigha/year)	Total Income Tk (Bigha/year)
1. Directly from importer	10	18.52	Disease	Insects	Weeds	800	1. Land preparation	3000	30000	Peak: 6 Off-peak: 3 Average: 4	27,200	1,20,000
			1800	1200	1200		2. Planting materials	15000				
2. Personal Preservation	38	70.37	**Total: 5000 Tk./Bigha/Year**				3. Weeding	1200				
							4. Irrigation	1500				
4. Farmers neighbor	3	5.56					5. Pest management	5000				
5. Seed Dealer	1	1.85					6. Picking flowers	500				
							7. Other cost	1000				
							**Total**	**27,200**				

During land preparation, farmyard manure is applied at a rate of 8000kg/ha, accompanied by Urea, TSP, MOP, Boron, and Zinc as basal doses. After pruning, additional applications of Phosphorus (P) and Potassium (K) are administered in conjunction with Nitrogen (N), Boron, and Zinc, applied at 20-day intervals. Emphasis on pest management is evident with a total annual cost of 5000 taka/bigha, encompassing disease management, insect pest control, weeding, and other protective measures.

Among the surveyed farmers, 44.44% sell tuberose flowers through middlemen, while 40.74% directly engage with businessmen and 12.12% sell to different companies. Production costs for tuberose cultivation remained relatively consistent across farmers, with the primary expenditure revolving around acquiring bulbs, the essential planting material, typically ranging from 1 to 2 Taka. This resulted in an initial cost of approximately 15,000 Tk/bigha for planting materials. Essential intercultural operations, including weeding, incurred an additional 1,200 Taka. Additionally, costs for watering, pesticides, and harvesting amounted to 1500, 5000, and 500 Taka/Bigha, respectively. Anticipated miscellaneous expenses, estimated at 1,000 Taka annually, were also taken into account. Farmers reported a total production cost of 27,200 Tk/Bigha.

The benefit-cost analysis for tuberose flower stick production was contingent on various factors. With an average production of 30,000 sticks per bigha annually, farmers received varying prices, averaging at 4 Tk/stick. During peak seasons, prices rose to 6 Tk/stick, while off-peak periods saw rates drop to 3 Tk/stick. Significant events for example Valentine’s Day and International Mother Language Day heightened demand, leading to increased prices. The total annual return from tuberose cultivation per bigha amounted to 120,000 Tk. Considering the total production cost of 27,200 Taka, approximately 92,800 Taka per Bigha was the annual net profit achieved from tuberose cultivation.

### Insights from farmers: Incidence, origins, and control measures for tuberose diseases

Approximately 29.63% of tuberose farmers attribute issues related to pests and diseases to bulbs, while 16.67% identify soil as a contributing source (Table 4). Challenges arising from local planting materials, imbalanced fertilizer use, and ineffective pesticides further compound pest and disease issues. Among the surveyed farmers, 64.81% primarily depend on pesticides to manage tuberose pests and diseases, considering it the most effective method. On the other hand, traditional methods are employed by 22.22%, bulb treatment by 18.52%, integrated pest management (IPM) by 9.26%, and other methods by 5.56%.

**Table 4:** Farmers’ opinion on the occurrence, source, controlling of tuberose diseases.

Sources of Tuberose Disease			Methods of disease control			Receiving Support and Services in Disease Control			Farmers’ opinion on better management options		
Sources	No. Respondents	Response (%)	Name of the methods	No. Respondents	Response (%)	Assistance/ Service	No. Respondents	Response (%)	Suggestions	No. Respondents	Response (%)
Bulb	16	29.63	Utilizing Pesticides	35	64.81	Visit by the field level officers	18	33.33	Use of healthy planting materials	24	44.44
Airborne	6	11.11	Employing Resistant Varieties	0	0	Experts suggestion	7	12.96	Effective use of pesticides	5	9.26
Soil-borne	9	16.67	Treatment of planting materials	10	18.52	Field day	5	9.26	Proper inter-culture operation	8	14.81
Local planting materials	7	12.96	Cultural practices & control measures	12	22.22	Field demonstration	4	7.41	Regular field visit	7	12.96
Farmers’ owned bulb	8	14.82	IPM method	5	9.26	Meeting with the field-level officers	17	31.48	More research on disease management	10	18.52
Use of imbalanced fertilizer	2	3.70	Others (if any)	3	5.56	No opinion	3	5.56	Assistance from DAE	8	14.81
Through irrigation water	1	1.85							Loan	5	9.26
Ineffectiveness of pesticides	3	5.56							Use of a resistant variety	2	3.03
Other sources (if any)	2	3.70									

Tuberose cultivators’ express positivity about receiving assistance, with 33.33% mentioning regular field visits by officers. Additionally, 31.48%, 12.96%, 9.26%, and 7.41% engage in meetings, listen to expert suggestions, attend field days, and observe demonstrations, respectively. A minority of 5.56% did not provide an opinion on this matter. Suggestions for enhanced disease management include a focus on healthy planting materials (44.44%). Other recommendations encompass increased research on disease management, proper intercultural operations, support from the Department of Agricultural Extension, efficient utilization of pest control chemicals, obtaining loans for cultivating flowers, and the utilization of immune varieties, among others.

### Insights from farmers: Ensuring safety, nutrient application, and administration of growth stimulants for tuberose plants

Based on feedback from 54 farmers, it is evident that the majority possess a considerable understanding of both plant protection and fertilization. Notably, neem oil emerged as the universally preferred substance (100%) for controlling various insect pests, while carbofuran had the lowest usage at 22.22% (Table 5). Among these, mancozeb was highly favored, with a usage rate of 92.59%, while chlorothalonil saw the least application at 27.78%. The recommended concentration for liquid fungicides and insecticides was approximately 2 ml/L of water, and for mancozeb, farmers employed a concentration of 3 gm/L of water for spraying.

**Table 5:** Farmers’ perspective on plant protection, fertilization, and growth regulators.

Categories	Usage	Product Group	Dose	No. of Respondents	Response (%)
Plant protection	Insecticides	Malathion	1 ml/l	50	92.59
		BHC dust	1.8 kg/ha	30	55.55
		Neem oil	3 ml/l	54	100
		Endosulphan	2 ml/l	22	40.74
		Imidachlorpid	1 ml/l	34	62.96
		Cypermethrin	2.5 ml/l	19	35.18
		Bifenthrin	5 ml/l	28	51.85
		Carbofuran	7–8 kg/ha	12	22.22
	Fungicides	Mancozeb	2 g/l	50	92.59
		Carbendazim	2 g/l	35	64.81
		Tricyclazole	2 g/l	44	81.48
		Chlorothalonil	1 g/l	15	27.78
		Iprodione	3 mg/l	31	57.41
		Difenoconazole	2 g/l	25	46.30
		Azoxystrobin	1 g/l	20	37.03
	Herbicides	Atrazine	3 kg/ha	35	64.81
		Oxyfluorfen	0.5 kg/ha	25	46.30
Fertilization	Fertilizers	Cow dung	8000 kg/ha	54	100
		Urea	125 kg/ha	54	100
		TSP	130 kg/ha	54	100
		MOP	150 kg/ha	54	100
		Boron	45 kg/ha	40	74.07
		Zinc	45 kg/ha	54	100
	PGR	GA3	150ppm/ha	35	64.81

Atrazine stood out as the most utilized herbicide, with a usage rate of 64.81%. According to farmers’ perspectives, optimal practices for disease control in tuberose cultivation involve the application of fertilizers. All farmers employed GA3 as a plant growth regulator and utilized cow dung, Urea, TSP, and MOP for fertilization. Additionally, a significant proportion of them (74.07%) incorporated boron into their fertilization practices.

## Discussion

A study was conducted in the Jashore district of Bangladesh for assessing economic and social standing among individuals cultivating tuberose, examine cultivation methods, and evaluate the prevalent diseases affecting cultivation. From 18 villages across four unions in Jhikargacha upazila, 54 farmers participated and underwent interviews using a questionnaire that had been tested beforehand. The questionnaire comprised three parts, focusing on the socio-economic background of the farmers, their employed production technologies, and their perspectives on tuberose diseases. The socio-economic aspect covered parameters such as the farmers’ gender, age, educational background, and patterns of land utilization. Within the cultivation method segment, aspects like fertilizer application, benefit-cost ratio, planting materials, and pest management were noted and subjected to analysis. Additionally, the perspectives of the farmers on various disease-related factors were considered, and their suggestions for improved disease management in tuberose cultivation were gathered.

According to a prior report, the primary regions for tuberose cultivation include Jashore, Chuadanga, Benapole, Singair, Tangail, Savar, Manikganj, and Gazipur. The findings indicate the expenses that associated with cultivating tuberose per hectare were calculated as Tk. 2,00,761 for total costs and Tk. 1,29,283 for variable costs. Most of the overall expenses, constituting 70%, were assigned to labor (30%), followed by land utilization (23%), and fertilizer (17%). Per hectare, the gross margin was recorded at Tk. 5,52,354, while the net return amounted to Tk. 4,80,876. The analysis of the production function showed that tuberose cultivation was positively impacted by human labor, seedlings, and irrigation [5].

A study conducted by Ahammed [9] focused on the socio-economic status of tuberose. The findings revealed notable differences in net marketing margins among various stakeholders. Retailers experienced the highest margin at Tk. 146.39, exceeding the figures for traders (Tk. 17.35), petty traders (Tk. 8.61), and wholesalers (Tk. 22.02). During the harvesting period, the producer’s per-unit selling price was Tk. 0.52, whereas the price paid by consumers was Tk. 0.74. In the post-harvest period, these figures increased to Tk. 2.49 and Tk. 2.63 for the producer’s per-unit selling price and consumer’s purchase price, respectively.

According to a preceding report, the composition of flower growers’ families exhibited diversity, with 28% having 1–4 members, 41.67% having 5–6 members, and 30% having more than six members. The breakdown by gender showed 93.33% being men, with 6.67% being women. Regarding education, within the sample of 60 farmers, it was observed that 25% lacked literacy, 28.33% had completed primary education, and 20% had finished secondary education. Additionally, 18.33% had education up to higher secondary education, and 8.33% had education beyond the higher secondary level among the farmers, indicating a noteworthy presence of education among the farmers. The calculated Benefit-Cost Ratio (BCR) for flower cultivation stood at 1.69, confirming the economic success of cultivating flowers in the examined region [10].

An investigation observed that the majority of flowers in Bangladesh are cultivated during the winter season. This is attributed to the prevalent practice of holding weddings in Bangladesh during the winter months, usually spanning from December through March. During the cold weather period, numerous flowers bloom, and there is a heightened demand for flowers due to various festivals. Consequently, both the demand for and supply of flowers are substantial during this period. The demand and pricing of flowers exhibit variations across different months, regular weekdays as well as special events throughout the year [11].

In their research on flower cultivation in Jashore; Islam, and Rahman [12] demonstrated that engaging in flower cultivation is a profitable venture, offering greater potential returns compared to other agricultural crops. Their study revealed a positive correlation between flower cultivation, national GDP growth, and employment generation.

Mou [7] conducted a thorough investigation in Bangladesh on the economic success of cultivating flowers and the distribution network. The study explored the cultivation and economic aspects of specific flowers in contrast to rival crops. The study recognized the flower marketing supply network and distribution channels of Bangladesh. Examined limitations in the marketing of flowers and put forward 22 suggestions with the goal of advancing the floral sector and strengthening its distribution system. The study employed a stratified unpredictable selection technique to collect primary data, including thirty-two farmers from Mymensingh, and twenty-one flower merchants, sellers, and distributors from various zones in Dhaka city. Gross margins per hectare were revealed in the findings: Tk. 1,359,824.20 for flowers and Tk. 46,362.14 for vegetables. In Dhaka City, the average marketing margins per 100 flowers for three intermediaries—BRAC, wholesaler-cum-retailer, and retailer—were Tk. 187.56, Tk. 638.39, and Tk.689.72, respectively. Major financial and technical challenges faced by flower farmers included the scarcity and high cost of mother stock, elevated prices of fertilizers as well as pesticides, absence of scientific expertise and education, and vulnerability to pest and disease attacks. Market-related problems included an insufficient and insufficient transport system as well as a telecommunication network, low market prices, insufficient market knowledge, and a limited unstructured market. The research also emphasized that individuals and contract farmers aged between 30 and 40 years were quick to adopt the system of flower farming under contract. About 21.87% of farmers under contract were identified as having no formal education. Education levels of growers varied, with 15.63% having up to primary education, 31.25% with secondary education, 25.00% with higher secondary education, and 6.25% with education beyond higher secondary levels. Moreover, about 59.38% of flower farming landowners integrated it with crop cultivation, while 3.12% combined it with service and business activities, and 21.88% participated within cultivating flowers alongside agricultural cultivation and business operations.

In their research, Bera and Majumder [13] highlighted the high costs associated with tuberose flower cultivation, primarily due to the substantial need for expensive human labor. However, they noted that if the necessary labor is readily available within the family, cultivating tuberose flowers can emerge as a profitable alternative to conventional paddy cultivation. Tuberose cultivation, in such cases, not only generates substantial income through family labor but also serves as a valuable source of employment in rural areas.

Previous reports investigated the export potential of specific flowers, including marigold, tuberose, gladiolus, and rose in Bangladesh. Utilizing a model showcasing exponential increase, the study analyzed the export trajectory of cut flowers as well as provided forecasts for future growth trends. Additionally, the report outlined the opportunities for expanding floriculture exports, emphasizing the comparative advantages derived from favorable climatic conditions that enable the country to tap into seasonal market opportunities in overseas markets [14].

Conducting a comprehensive study, the researcher assessed the current state of flower cultivation and identified the challenges encountered by farmers in Jhikargacha Upazila, Jashore district, Bangladesh. The investigation involved collecting data on various aspects of flower cultivation, such as the types of flowers grown, timing of cultivation, methods for obtaining seeds and seedlings, propagation practices, fertilizer, and manure usage, as well as issues related to diseases, insect pests, and intercultural operations. The findings highlighted that the most widespread and severe problem faced by farmers was the lack of access to loan facilities. Other commonly encountered challenges included insufficient preservation facilities, low market prices, and the high cost of fertilizers and pesticides [15].

The findings of this survey study provide valuable insights into the cultivation practices, challenges, and perspectives of tuberose farmers in the Jashore district of Bangladesh. Through a comprehensive analysis of survey data, several key themes emerged, shedding light on various aspects of tuberose farming in the region.

Firstly, the study revealed the prevalent cultivation practices among tuberose farmers in the Jashore district of Bangladesh. It was found that a majority of farmers adopt traditional farming methods, including manual labor for planting, weeding, and harvesting. Additionally, most farmers rely on chemical fertilizers and pesticides for crop management, highlighting the need for greater awareness and adoption of sustainable farming practices.

Furthermore, the survey highlighted the challenges faced by tuberose farmers in Jashore district. These challenges include limited access to agricultural inputs, such as quality planting materials and irrigation facilities, as well as fluctuating market prices for tuberose flowers. Additionally, farmers expressed concerns about pest and disease management, with many relying heavily on chemical pesticides to combat these issues.

The perspectives of tuberose farmers on various aspects of farming were also explored in the study. Farmers expressed a strong desire for access to training and extension services to improve their knowledge and skills in tuberose cultivation. Moreover, there was a notable interest among farmers in adopting sustainable farming practices, such as organic fertilization and integrated pest management, to reduce reliance on chemical inputs and mitigate environmental risks.

In addition to addressing challenges specific to tuberose farming, the study also uncovered broader issues affecting agricultural sustainability in the region. Climate change emerged as a significant concern among farmers, with changing weather patterns impacting crop yields and increasing the frequency of pest outbreaks. As such, there is a pressing need for climate-resilient agricultural practices to mitigate these risks and ensure the long-term viability of tuberose farming in Jashore district.

Overall, this survey study provides valuable insights into the current state of tuberose farming in the Jashore district of Bangladesh. The findings underscore the importance of adopting sustainable farming practices, enhancing access to agricultural resources and training, and addressing the challenges posed by climate change to ensure the continued prosperity of tuberose farmers in the region.

## Conclusions

In summary, this in-depth analysis has provided a comprehensive understanding of the socio-economic dynamics shaping tuberose farming in the Jashore district of Bangladesh. Through a careful examination of cultivation practices, economic indicators, and social factors, this study has shed light on the complexities and challenges faced by tuberose farmers in the region. By uncovering the intricate interplay between economic constraints, social structures, and agricultural practices, the research has identified key areas for intervention and improvement. From addressing barriers to education and training to enhancing access to markets and resources, there exist tangible opportunities to cultivate positive change within the tuberose farming community in Jashore district. Moreover, the findings underscore the importance of tailored strategies that take into account the unique socio-economic context of the region. By fostering collaboration between stakeholders, including farmers, policymakers, and agricultural experts, we can work towards sustainable solutions that promote economic prosperity, social equity, and environmental stewardship within the tuberose farming sector. As we embark on this journey of change, it is imperative to prioritize inclusivity, resilience, and innovation. By harnessing the collective wisdom and expertise of all stakeholders, we can cultivate a brighter future for tuberose farming in the Jashore district, ensuring that it remains a vital pillar of economic growth and community development in Bangladesh.
